# Effect of Chromium on Microstructure and Oxidation Wear Behavior of High-Boron High-Speed Steel at Elevated Temperatures

**DOI:** 10.3390/ma15020557

**Published:** 2022-01-12

**Authors:** Pengjia Guo, Shengqiang Ma, Ming Jiao, Ping Lv, Jiandong Xing, Liujie Xu, Zhifu Huang

**Affiliations:** 1 National Joint Engineering Research Center for Abrasion Control and Molding of Metal Materials, Henan University of Science and Technology, Luoyang 471000, China; guopengjia@stu.xjtu.edu.cn (P.G.); wmxlj@126.com (L.X.); 2 State Key Laboratory for Mechanical Behavior of Materials, School of Materials Science and Engineering, Xi’an Jiaotong University, Xi’an 710049, China; mengxiaoli@stu.xjtu.edu.cn (M.J.); lvping2020@stu.xjtu.edu.cn (P.L.); jdxing@mail.xjtu.edu.cn (J.X.); hzf@mail.xjtu.edu.cn (Z.H.)

**Keywords:** high-boron high-speed steel, high-temperature wear, oxidation, microstructure, wear mechanism

## Abstract

In order to investigate the effect of Cr content on the microstructures and oxidation wear properties of high-boron high-speed steel (HBHSS), so as to explore oxidation wear resistant materials (e.g., hot rollers), a scanning electron microscope, an X-ray diffractometer, an electron probe X-ray microanalysis and an oxidation wear test at elevated temperatures were employed to investigate worn surfaces and worn layers. The results showed that the addition of Cr resulted in the transformation of martensite into ferrite and pearlite, while the size of the grid morphology of borides in HBHSSs was refined. After oxidation wear, oxide scales were formed and the high-temperature oxidation wear resistance of HBHSSs was gradually improved with increased additions of Cr. Meanwhile, an interaction between temperature and load in HBHSSs during oxidation wear occurred, and the temperature had more influence on the oxidation wear properties of HBHSSs. SEM observations indicated that a uniform and compact oxide film of HBHSSs in the worn surface at elevated temperatures was generated on the worn surface, and the addition of Cr also reduced the thickness of oxides and inhibited the spallation of worn layers, which was attributed to improvements in microhardness and oxidation resistance of the matrix in HBHSSs. A synergistic effect of temperature and load in HBHSSs with various Cr additions may dominate the oxidation wear process and the formation and spallation of oxide films.

## 1. Introduction

In many high-temperature manufacturing fields, the rollers and tools acting as forming or molding equipment are of importance in the production of parts and components. The consumption of these rolls and tools increases owing to the wear and oxidation of the equipment at rigorous conditions and elevated temperatures. Meanwhile, the heavy wear loss and frequent failure of these components have detrimental results for service safety, reliability of the machines and efficiency [1,2,3]. Until recently, semi-steel, infinite chill-cast iron, high chromium cast iron and high-speed steel were widely applied in the production of rollers [4,5,6]. For a long time, high-chromium cast iron was considered the best wear-resistant material [6,7]. Thereafter, a large number of MC-type carbides were found in high-vanadium high-speed steel to achieve further improvements in performance in hardness, thermal stability and wear resistance [8,9,10]. However, some disadvantages were discovered with the utilization of high vanadium high-speed steel rolls under extremely demanding working conditions. For instance, the production cost grows because of the copious addition of expensive alloying elements such as tungsten, vanadium and molybdenum. Moreover, the toughness and thermal fatigue properties of the alloys are insufficient due to the precipitation of coarse hard and brittle carbides [6,11]. Consequently, it is practically important for industrial applications to develop new roll materials with low cost and outstanding wear resistance [6].

Recently, much attention has been paid to high-boron high-speed steel (HBHSS), which is regarded as a desired roll material due to its tailorable and controllable combination properties such as high hardness and thermal stability, improved fracture toughness and excellent wear resistance [12,13,14,15,16]. For instance, Ren et al. investigated the microstructures and impact toughness of HBHSS (e.g., with chemical composition of Fe-2.0 wt.% B-0.4 wt.% C-6.0 wt.% Cr-4.0 wt.% Mo-2.0 wt.% Al-1.0 wt.% Si-1.0 wt.% V-0.5 wt.% Mn-0.1 wt.% Ca-0.6 wt.% Ti), showing that it could be improved owing to the active Ca-Ti element inhibition growth for borocarbides and subsequent refinement of borides [12]. Fu et al., studied the thermodynamics and microstructural evolution of HBHSSs with various aluminum additions to indicate the formation of δ-Fe changing into α-Fe and the formation of ferrite due to the excessive addition of aluminum in HBHSS [13]. Yuan et al. examined the microstructure and high-temperature tribology behavior of high-boron high-speed steel under different heat treatments, and the results pointed out that increasing the quenching temperature could reduce the high-temperature friction coefficient at 500 °C friction (e.g., the smallest value of 0.425) [14]. Moreover, Cen et al. showed that heat treatment could effectively improve the bulk hardness of HBHSS in casting and centrifugal cast processes [15,16]. Meanwhile, Ma et al. also revealed that adding aluminum into HBHSSs could refine the microstructures and promote dispersive precipitations in HBHSSs [17,18]. Therefore, HBHSSs (e.g., mainly containing about 0.4 wt.% c and 1.5–2.0 wt.%B as well as 4.5–6.0 wt.%Cr and a little other alloying elements less than 2 wt.%) have been extensively investigated as roll materials owing to their low cost and improved performance.

In addition, the unique microstructures and properties of Fe-B-C alloy can be easily regulated by a wide-range adjustment of the high-boron and carbon contents to control the hard phase Fe_2_B and the metal matrix [19,20,21,22]. The excellent wear performance of Fe-B alloy is mainly attributed to its microstructure, as reported in previous work [22,23,24,25,26]. Novel Zr-alloyed high-boron steels with 3.2 wt.% B and 6 wt.% Zr as a corrosion-resistant stainless steel used in nuclear power plants exhibited better corrosion resistance to aggressive chloride ion-contained environments to guarantee the safety and efficiency of spent nuclear fuel storage [27], while the boron content in stainless steels can also improve resistance to solidification cracking in the fusion zone during the welding process [28]. Therefore, Fe-B alloys or high-boron steels can be well designed with different chemical compositions and solidification conditions to control different performance requirements and service conditions.

Normally, high-temperature oxidation of wear-resistant alloys occurs, which may affect the wear property and failure mechanism of the alloys. Generally, oxidation occurs on alloys under high temperatures, which affects the wear property [6,29,30]. The wear mechanism of the roller materials under high temperatures performs the combination of adhesive spallation, micro-cutting and serious oxidation [31,32]. Therefore, Cr and Al elements often are added into the alloys or steels to improve the oxidation wear properties owing to improvements in the hardness and the formation of protective oxides. Investigations indicated that the microstructure and mechanical properties of Fe-B-C alloy could be ameliorated by the appropriate addition of chromium [33,34]. According to Xiao’s computational reports [35,36], transition metal elements (e.g., chromium, molybdenum and manganese) can replace iron atoms in Fe_2_B crystals to improve the hardness and toughness of Fe_2_B, simultaneously using the first principle calculations based on density functional theory according to band theory and the chemical bond strength of Stoner’s polarization theory. Jian et al. [23,37,38] studied the effect of chromium on the improvements in the mechanical properties and two-body wear performance of Fe-3.0 wt.% B alloy. Their results showed that the appropriate addition of chromium could improve the two-body wear resistance of boron-containing alloys [39]. However, high-temperature oxidation wear occurs in many applications, and research on the effect of Cr in high-boron iron-based alloys at a high-temperature oxidation wear condition is scarce. Therefore, it is significant and urgent to study the effect of Cr on the oxidation wear properties of high-boron high-speed steel at high temperatures so as to understand the harsh wear behaviors and design better roll material.

In this work, the specific effect and wear mechanism of Cr on the oxidation wear performance and worn morphologies of high-boron high-speed steel at high temperatures were investigated. Additionally, the comprehensive influences of different temperatures and applied loads on oxidation wear were discussed to clarify the synergistic effects of microstructures and wear conditions on the high-temperature wear behavior and related wear mechanism of HBHSSs.

## 2. Materials and Methods

### 2.1. Sample Preparation

In the present paper, the chemical composition of the investigated high-boron high-speed steel (HBHSS) is listed in Table 1, as designed in Refs. [12,13,14,15,16,17,18], indicating the so-called HBHSS. The four samples with different Cr contents are denoted as A1, A2, A3 and A4 samples of HBHSSs, and M2 high-speed steel is used as a comparative sample to qualitatively characterize the properties of HBHSSs. The HBHSSs were prepared in a 10 kg intermediate frequency-induction melting furnace (Xi’an Yinhai Electric Furance Co., Ltd., Xi’an, China). The pure iron and pig iron were melted first, and then ferromanganese, ferrochromium, ferrosilicon, ferrotungsten and ferrovanadium were added into the furnace in that sequence. When all the materials had completely melted, the preheated raw ferroboron and ferrotitanium materials were added into the furnace after a little pure aluminum, to deoxidize, was also added to finally obtain the desired HBHSSs.

### 2.2. High Temperature Wear Experiment

**Figure 1:**
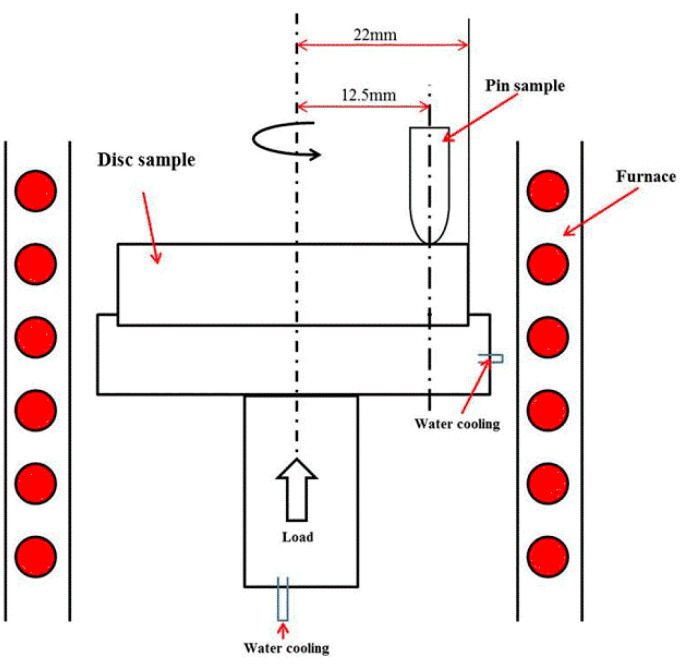
Schematic diagram of the set-up of the oxidation wear experiment.

The oxidation wear experiments were carried out on an MMG-500 high-temperature wear tester (Jinan Hansen Precision Instrument Co., Ltd., Jinan, China). The maximum test force of this tester was 500 N, and the temperature of the tester can be altered from room temperature to 1100 °C. The high-temperature wear testing method was performed in the pin-on-disc friction pair mode. All samples of HBHSSs were fabricated into standard specimens with the dimensions of 5 mm in diameter and 12 mm in length as the pin sample. The disc sample was the Inconel 718 nickel base superalloy with a diameter of φ 44 mm. The set-up of the oxidation wear experiment is shown in Figure 1.

After testing, the samples were polished, subjected to pre-sliding friction for 5 min to ensure the close fit of the friction surface, and then placed in alcohol for ultrasonic cleaning treatment. After drying, the original mean weight *m*_0_ was collected by the average value taken from the 12-time accurate measurement using an electron balance, and the actual volume *V*_0_ of the pre-sliding sample was also obtained by the drainage method [40]. Thereafter, the pre-sliding pin and disc samples mentioned above were carefully installed on the wear tester for the oxidation wear tests. After the tests, the weight change *m*_1_ was recorded to obtain the weight loss data. The weight losses of all tested samples under the given temperatures and loads were the average values of three homogeneous testing specimens. The wear test parameters are shown in Table 2.

The high-temperature wear rate calculated by the formula is given as follows [6]:

(1) $$w=\frac{\Delta W}{LP}$$

Here, *w* is the wear rate and Δ*W* represents the weight loss of the pin sample after the wear experiment. *L* is the wear distance of the pin sample and *P* is the load of the pin sample on the disc sample.

All the tested samples were ground and polished, and then etched by 4 vol% (volume ratio) nitrate alcohol solution to observe the microstructures. Microanalysis of the specimens was carried out using a scanning electron microscope (SEM, VEGAII XMUINCA, TESCAN, Brno, Czech Republic) with a back-scattered electron image (BEI) and a secondary electron image (SEI) system, X-ray diffraction (XRD, D/Max-2400X, Rigaku Corporation, Tokyo, Japan), an electron probe micro-analyzer (EPMA, JXA-8230, Japan electron optics laboratory Co., Ltd., Tokyo, Japan) equipped with wavelength dispersive X-ray (WDX) analysis and energy-dispersive X-ray (EDX) spectroscopy to identify microstructures before and after oxidation wear of HBHSSs for the microstructural characterization. The XRD was performed using Cu-Kα radiation coupled to continuous scanning at 40 kV and 200 mA as an X-ray source. The as-polished specimens for XRD were scanned in the angle 2θ, ranging from 20° to 100°, with a step size of 0.02° and a collection time of 10 s. Bulk hardness was measured on an HR-150A Rockwell hardness tester(HR-150A, Beijing Shidai Shanfeng Technology Co., Ltd., Beijing, China), and the microhardness of the matrix was measured using an HXD-type 1000 Vickers-hardness tester(HXD-1000TMC, Shanghai Taiming Optical Instrument Co., Ltd., Shanghai, China) with a load of 50 gf. The volume fractions of various phases in HBHSSs were obtained using Leica Qwin 5.0 image analysis (OM, Leica DMI5000M, Leica, Wetzlar, Germany) with a microscope at 12 randomly chosen fields of view to statistically collect the volume fraction.

## 3. Results and Discussion

### 3.1. As-Cast Microstructure of HBHSSs with Various Cr Additions

**Figure 2:**
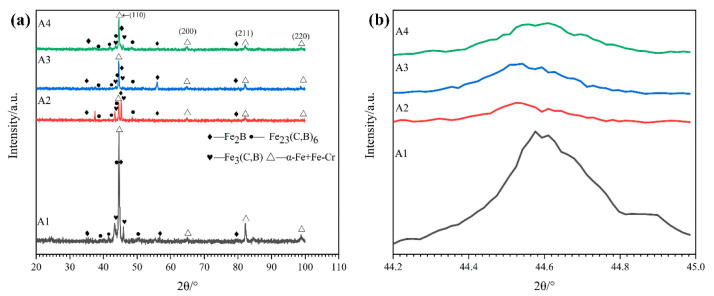
XRD patterns of the as-cast high-boron high-speed steel: (**a**) XRD pattern, (**b**) local peaks of Fe (110).

The XRD patterns of the as-cast high-boron high-speed steels with different Cr contents are shown in Figure 2. It can be seen that as-cast microstructures of the alloys with 4.80 wt.% Cr are mainly composed of M_2_B (M = Fe,Cr), Fe_3_ (C,B), Fe_23_ (C,B)_6_, α-Fe and Fe-Cr (i.e., martensite matrix), whereas the peaks of the Fe-Cr matrix gradually disappear and the peaks of α-Fe increase. This means that Cr may promote the formation of ferrite. Additionally, the peaks of M_2_B borides gradually shift to the left with the increase in Cr in HBHSS, which suggests that the Fe-rich M_2_B boride (e.g., (Fe,Cr)_2_B) transforms into Cr-rich M_2_B boride (i.e., (Cr_1_._65_Fe_0_._35_B_0_._96_ or CrFeB boride) [17,24,33]. The reason for this is that the atomic radii of Cr and Fe are close, and it is easy to form a substitutional solid solution of an Fe atom replaced by a Cr atom in the M_2_B lattice when the Cr content exceeds 8.8 wt.% in the HBHSSs [24,33,34].

**Figure 3:**
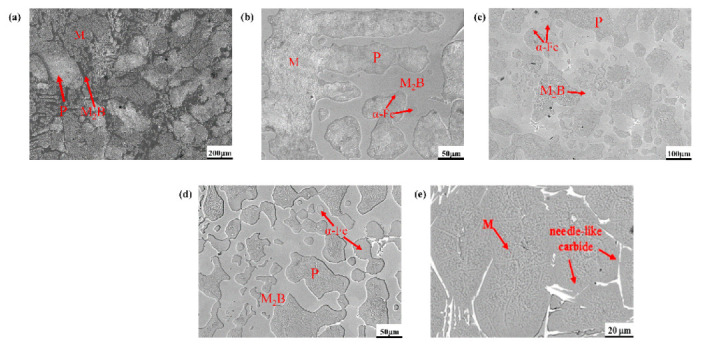
SEM image of high-boron high-speed steel: (**a**) A1, (**b**) A2, (**c**) A3, (**d**) A4, (**e**) M2.

Figure 3 shows the as-cast microstructures of high-boron high-speed steel with different Cr contents and M2 high-speed steel. From Figure 3a–d, it is clearly seen that the microstructures of the A1 sample are mainly composed of martensite, pearlite and some interdendritic continuous netlike and fish-bone M_2_B borides (M = Fe, Cr, Mo, W, etc). Basically, the metal matrix mainly consists of martensite [17,18]. With the increase in Cr content, the martensite matrix gradually transforms into much pearlite and some ferrite, while the morphology of netlike M_2_B borides increasingly changes into continuous blocky and fenstral fabrics; namely, the grid morphology of boride is distributed over the matrix, as shown in Figure 3b. Successively, the amount of M_2_B increases with increasing Cr content (Figure 3b,c). At the same time, the martensite seems to disappear, while some ferrite occurs and stays at the grain boundary of pearlite and eutectic borides, which reveals a circle structure around the borides to annularly package M_2_B compounds. It may be beneficial for the brittle boride to resist external loads for the inhibition of cracking. Additionally, the high Cr content may refine the size of the grid morphology of boride, as shown in Figure 3b–d. Obviously, the Cr content promotes the formation of ferrite, which may be attributed to the substitutional solid solution of Cr to enlarge the ferrite zone, and also to replace some Fe atoms of the M_2_B lattice structure [24,34]. Compared with HBHSSs, M2 high-speed steel comprises the martensite matrix and M_7_C_3_ carbides, as well as some MC carbides, as shown in Figure 3e.

**Figure 4:**
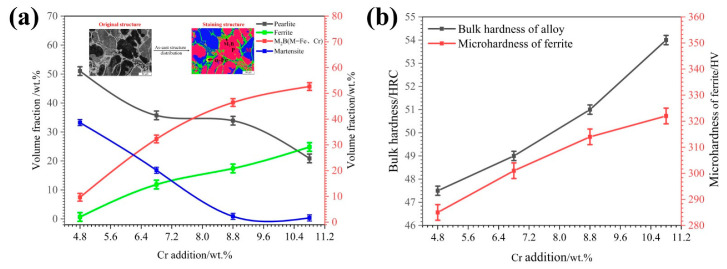
Volume fraction of various microstructures in HBHSSs and bulk hardness, as well as ferrite microhardness:(**a**) volume fraction vs. Cr content, (**b**) the bulk hardness and microhardness of ferrite matrix vs. Cr content.

Figure 4 shows the volume fractions of various microstructures and the hardness of HBHSSs with different Cr contents. From Figure 4a, it is clear that the volume fractions of the M_2_B (M = Fe, Cr) and ferrite may be constantly enhanced with the increase in Cr content, while the volume fractions of martensite and pearlite display a continuous decrease, especially for the amount of martensite. As a matter of fact, the addition of Cr may shift the eutectic point of Fe-Cr-B to the left and promote the formation of a more eutectic M_2_B structure during the solidification process. Additionally, the solid solution of some Cr in the Fe matrix may reduce the martensite and result in lots of ferrites. From Figure 4b, with the increase in Cr content, the bulk hardness of the HBHSSs and the microhardness of the ferrite can gradually increase owing to the increasing amounts of Cr in both M_2_B borides and the matrix [33,34]. Obviously, the addition of the Cr element to HBHSSs may stabilize eutectic M_2_B or (Fe, Cr)_2_B under the current Cr contents (namely, less than 11 wt.% Cr in the steels).

### 3.2. High-Temperature Oxidation Wear Rates of HBHSSs

**Figure 5:**
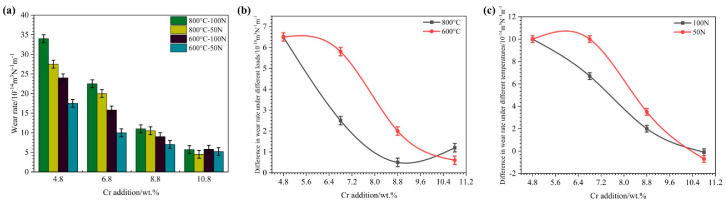
High-temperature oxidation wear rates of HBHSSs with various Cr contents at different testing temperatures and applied loads: (**a**) oxidation wear rates under different loads at 800 °C and 600 °C vs. Cr addition; (**b**) different values of oxidation wear rate at 100 N and 50 N loads vs. Cr addition; (**c**) different values of oxidation wear rate at 600 °C and 800 °C temperatures vs. Cr addition.

Figure 5 shows the high-temperature oxidation wear rates of HBHSSs with various Cr contents at different testing temperatures and applied loads. From Figure 5a, it is clear that the oxidation wear rates of HBHSSs dramatically decrease with the increase in Cr contents. Obviously, when the Cr content is less than 8.80 wt.%, the oxidation wear rate of HBHSSs with the same Cr content varies greatly under different temperatures and applied loads, whereas the oxidation wear rates of HBHSSs with high Cr contents (i.e., 8.80 wt.% and 10.80 wt.% Cr in HBHSS) are slightly altered. Clearly, the addition of Cr can strongly inhibit the oxidation wear rate. Meanwhile, the higher temperatures and large applied loads can distinctly increase the oxidation wear rate. The reasons for this are that, on the one hand, the alloying Cr may not only improve bulk hardness (e.g., the microhardness of the matrix and borides) but also enhance the solid solution amount of Cr in the matrix and oxidation resistance [6,9,12,14]. On the other hand, the loads and temperatures can improve the intensity of wear damage and oxidation, as well as the synergistic effects of oxidation and wear. Therefore, the wear rate at 800 °C under the same Cr content and load is basically higher than 600 °C, thus indicating that the increasing temperature will hinder the high-temperature oxidation wear resistance of HBHSSs. In addition, the oxidation wear rate also increases obviously with the increase in the load [6,9,12,14].

The oxidation wear rate difference values of HBHSSs under different oxidation wear temperatures and loads are depicted in Figure 5b,c. The different values of the oxidation wear rate under various loads (e.g., loading at 50 N and 100 N) at 600 °C decreases sharply when the variation in Cr content in HBHSSs increases from 6.8 wt.% to 8.8 wt.%, while it reduces gently at low Cr and high Cr additions (Figure 5b). However, the difference value of the oxidation wear rate under various loads (e.g., loading at 50 N and 100 N) at 800 °C dramatically declines when the content of Cr in HBHSSs does not exceed 8.8 wt.%, whereas it slightly rises when the Cr content is 10.8 wt.%. The reason for this is mainly that the addition of Cr can enhance both the hardness of steels and the oxidation resistance of the matrix. Furthermore, it is worth noting that the different values of the oxidation wear rate for HBHSSs with increasing Cr content under various temperatures (e.g., at 600 °C and 800 °C) descend slowly first and then rapidly when the oxidation wear loads are 50 N and 100 N, respectively, as shown in Figure 5c. Apparently, there should be a distinct synergistic effect of the temperature and load changes for the oxidation wear resistance of HBHSSs with various Cr contents. That is to say, oxidation wear may be strongly affected by the hardness and oxidation resistance of HBHSSs through the adjustment of Cr content, which may be closely related to the oxidation of worn surface films as well as their microstructures.

### 3.3. Surface and Cross-Sectional Morphologies of HBHSSs

#### 3.3.1. Surface Morphologies of HBHSSs

**Figure 6:**
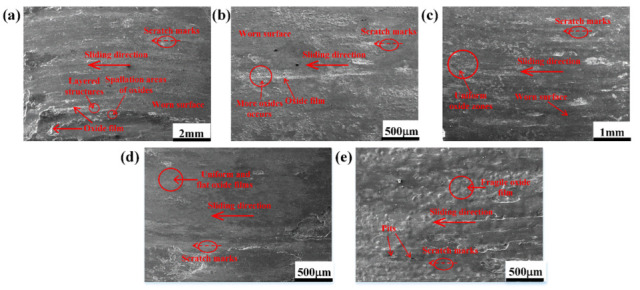
Surface morphologies of HBHSSs at 800 °C for 50 N: (**a**) A1, (**b**) A2, (**c**) A3, (**d**) A4, (**e**) M2.

Figure 6 shows the surface morphologies of the HBHSSs as the pin samples after oxidation wear. As shown in Figure 6a, the entire oxidation worn surface of HBHSSs with a low Cr content at 800 °C under an applied load of 50 N displays most of the gray areas, with a small number of light zones. Obviously, the gray areas are some of the worn surface while the light zones lie in the oxidation zone and actually belong to the oxide films interspersed on the worn surface. The traces of a typical wear scratch along the wear direction are observed, and they are parallel to the sliding direction; some oxide films also occur and accumulate at the worn surface, especially in the ridges adjacent to the ploughing. Moreover, the uplift edge of the friction wear in the A1 sample undergoing oxidation wear at 800 °C for 50 N appears as layered structures and some spallation areas of the oxides, which indicates that oxidation occurs during the high-temperature wear process and that the oxides may be strongly ploughed to easily deform and crack under the friction force, acting as a light transition layer of oxide film. As shown in Figure 6b,c, the light and white oxides in the worn surface of the A2 sample gradually increase, while the sliding depth and amount of the ploughing and furrow traces become shallower, smaller and discrete. Clearly, the superficial friction ploughing areas cannot be easily distinguished from the transition layer of oxide film mutually. The reason for this is that the friction wear damage of HBHSSs with higher Cr contents (i.e., A2 and A3 samples with 6.80 and 8.80 wt.% Cr, respectively) and the oxidation resistance can be improved to create more uniform oxide zones on the worn surface. As shown in Figure 6d, when the Cr content is 10.8 wt.%, the wear scar completely disappears, and the entire worn surface of HBHSS at 800 °C for 50 N is covered by uniform and flat oxide films without fraction trances. Obviously, the worn surface roughness of the A4 sample is greatly improved. In comparison with HBHSSs, the oxidation of the worn surface in M2 high-speed steel shown in Figure 6e is fairly rough, and the oxide film formed on it is becomes fragile and scattered. Clearly, there are as many spalling pits and craters of the worn surface in M2 as on the worn surface films, which cannot effectively protect the alloy. Therefore, from the results in Figure 6, high-boron high-speed steel at 800°C shows better oxidation wear resistance than M2 high-speed steel, and only a little oxide may peel off the worn surface with slight sliding scars and ploughing scratches in the HBHSSs. The oxidation wear layer is thick and tightly bonded with the substrate of HBHSSs, which greatly protects the HBHSSs from directly participating in the oxidation wear; instead, the combined effects between the microstructure and environmental interaction prevent wear and oxidation. Meanwhile, the addition of Cr can strongly consolidate the oxidation wear resistance of HBHSSs, especially for higher temperatures and larger loads.

**Figure 7:**
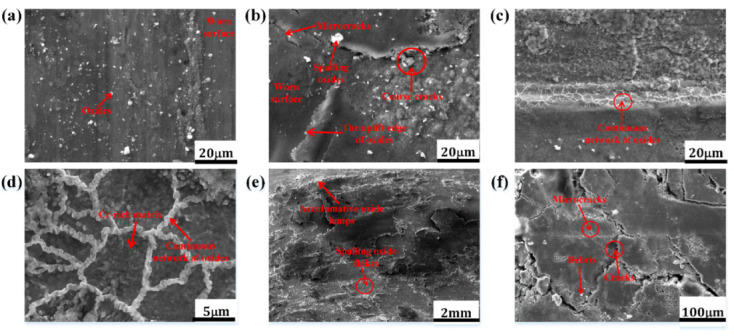
Surface morphologies of pin specimens and disc specimens for 50 N loads: (**a**) pin sample with 4.8 wt.% Cr at 600 °C for 50 N; (**b**) pin sample with 4.8 wt.% Cr at 800 °C for 50 N; (**c**) oxidation zone of pin sample at 800 °C for 50 N; (**d**) high-magnification oxidation zone of pin sample at 800 °C for 50 N; (**e**) surface morphologies of disc sample at low-magnification; (**f**) surface morphologies of disc sample at high magnification.

Figure 7 shows the high-magnification worn surface morphologies of HBHSS with 4.8 wt.% Cr at 50 N loads. As can be seen in Figure 7a, the surface wear morphology at 600 °C is relatively integrated, smooth and flat, in which the worn surface is displayed with a few shallow furrow traces. Meanwhile, the whole worn surface is gray and only a few white and bright oxides are formed on it. However, at the worn surface of 800 °C, some obvious coarse cracks and oxidation zones can be observed in Figure 7b. Clearly, some of the oxides can peel off from the worn surface of the HBHSSs. In fact, the original oxides at the bottom of the cutting grooves and ploughing regions for the pin samples are removed and peeled off due to the presence of the fraction loads. Obviously, some friction scratches and distinct oxidation films were overlaid on the surface, and there is a continuous network of white bright oxides regenerated around the Cr-rich eutectic boride region, as shown in Figure 7c. Although the oxides preferentially and favorably formed at the boundaries of matrix and borides, they may gradually crack and break during the actions of wear loads and a mismatch of multicomponent oxides under thermal stress, thus leading to the aggravating wear. A further magnification of the oxidation zone in the worn surface at 800 °C for 50 N is shown in Figure 7d. We found that there was a continuous network of regenerated white bright oxides, which occurs around the boundary zone of the Cr-rich matrix and the Cr-rich eutectic boride. The netlike oxide films indicate that the high temperature may strongly aggravate oxidation and promote the formation of oxide films during the oxidation wear process (e.g., Figure 7c,d) [6,7,8,9,12,14]. Figure 7e,f show the surface morphology of the rubbing disc sample (e.g., Inconel 718). In comparison with the pin sample, there are a large number of thick oxides accumulated and piled on the worn surface of the disc sample, which exhibits loose oxidation morphologies in the form of oxide lumps and spalling flakes with different sizes at the worn surface. Additionally, some large and deep ploughing and groove marks exist through the oxide films, as shown in Figure 7e. The magnification image in Figure 7f clearly reveals that many deeper and larger microcracks and fragments or debris adjacent to the cracking zone can be observed on the surface of the massive oxides (Figure 7f), which also implies that these superficial oxides are very fragile and do not adhere well to the substrate of the disc because of the friction wear forces and appearance of the non-protective oxide films; this leads to some wear debris participating in the oxidation wear process and acting as abrasive particles.

#### 3.3.2. Cross-Sectional Morphologies of HBHSSs

**Figure 8:**
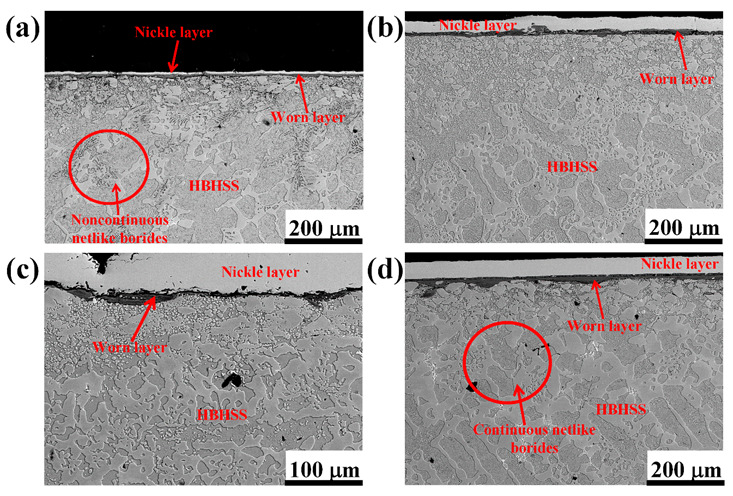
Cross-section morphologies of HBHSSs after oxidation wear at 800 °C for 100 N: (**a**) A1, (**b**) A2, (**c**) A3, (**d**) A4.

Figure 8 shows the cross-sectional morphologies of pin samples for HBHSSs with various Cr additions at 800 °C for 100 N. After oxidation wear, the worn surface was coated with Ni-planting, and the cross-sectional morphologies were carefully prepared, as depicted in Figure 8. The results indicate that the high-temperature oxidation wear resistance of the HBHSSs gradually increases with increase additions of Cr. The thickness of the oxidation wear accumulation layer of the HBHSSs is enhanced with increasing additions of Cr, whereas inner oxidation layers decrease in thickness and become continuous and compact. The cause of the changes is that Cr additions can consolidate hardness and corrosion resistance. Interestingly, the microstructural changes in the subsurface at the bottom of the oxidation wear layer take place. The fragmentation of some borides in HBHSSs constantly occurs with increased additions of Cr. Additionally, part of the secondary precipitates seems to be generated from the matrix of the substrate, which may likely be attributed to the degree of decomposition from the solid solution of substitutional Cr, both in the matrix and in borides, during oxidation wear at high temperatures [6,9,12,14,41]. Similarly, the presence of oxide scales acting as a tribolayer can be responsible for the improved cracking resistance of the annealed SPS samples, and wear behavior is influenced by the precipitates of intermetallic phases strongly affecting hardness [41], which is caused by changes in the borides in HBHSSs during the oxidation wear process. Meanwhile, compared with the low additions of Cr in Figure 8a,b, the continuous netlike and refined morphologies of borides in HBHSSs with increased levels of Cr may play a key role in supporting and blocking effects to resist wear and oxidation damages; this exhibits a beneficial combination of actions, such as oxidation wear resistance, to improve the oxidation wear resistance of HBHSSs with high additions of Cr.

**Figure 9:**
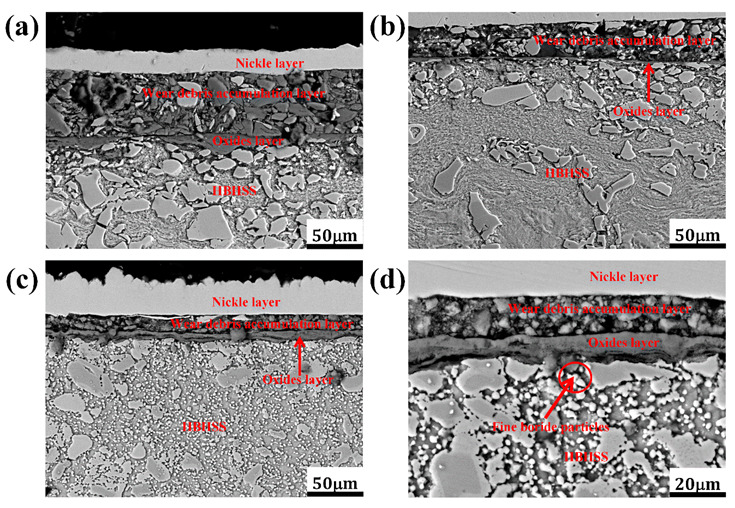
Cross-section morphologies of HBHSS with 4.8 wt.%Cr after oxidation wear at different temperatures and loads: (**a**) at 600 °C for 50 N, (**b**) at 600 °C for 100 N, (**c**) low magnification at 800 °C for 50 N, (**d**) high magnification at 800 °C for 50 N.

Figure 9 shows the oxidation wear cross-section morphologies of the 4.8 wt.% Cr sample under different temperatures and loads. From Figure 9a, it can clearly be seen that the oxidation wear layer has been delaminated. The white and bright part on the top is the nickel-plated layer. In the covering layer of the friction and wear surface, the first layer overlaid on the top is composed of some broken particles of wear debris, and there are large quantities of friction and wear debris accumulated on the worn surface between the oxide layer and the nickel-plated layer (i.e., the first layer on top of the worn surface). Meanwhile, a continuous and dense oxide layer is attached to the metal matrix with a dark color in the layers of Figure 9. Obviously, due to the presence of the oxide layer, the matrix of HBHSSs is not in direct contact with the disc sample, which means that the weight loss of oxidation wear mainly results from the weight loss of surface oxide spalling and fragmentation. From Figure 9b, with the increase in the applied load, the thickness of the oxide layer gradually decreases. Obviously, these results indicate that the oxides are more likely to undergo plastic deformation as the applied load increases. Additionally, part of the oxides peels off, which demonstrates that the anti-wear performance becomes worse due to the higher loads at high-temperature wear [6,9,12,14,16]. From Figure 9c,d, in the low Cr sample, the morphology of the cross-sectional oxide layer is still relatively intact and unbroken at high temperatures, and no cracking occurs. Clearly, it is tightly combined with the metal matrix of HBHSSs. It can be seen from Figure 9d that most of the eutectic borides adjacent to the worn layers become very fine particles in the matrix, and the eutectic borides close to the surface area are no longer continuous, which demonstrates that the supporting effect of subsurface borides in the HBHSSs on the surface oxide layer is reduced and weakened, thus further aggravating wear damage under higher temperatures and loads.

### 3.4. Microstructures and Compositions of Oxide Film Detected by XRD and EPMA

**Figure 10:**
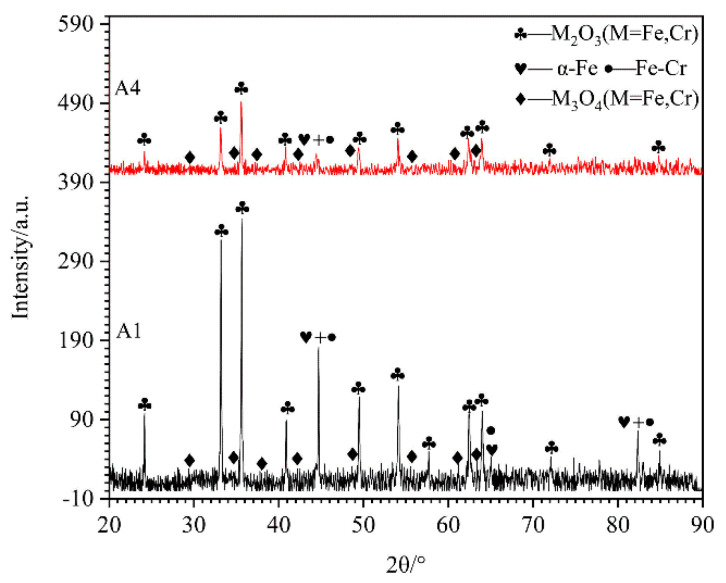
XRD patterns of A1 and A4 samples after oxidation wear at 800 °C for 100 N.

Figure 10 shows the XRD patterns on the surfaces of HBHSSs tested at 800 °C for 100 N, namely A1 and A4 samples. The surface oxides of the A1 sample are composed of M_2_O_3_ (M = Fe, Cr), M_3_O_4_ (M = Fe, Cr) and α-Fe, while there is a little diffraction peak of α-Fe on the surface of A4 and only M_2_O_3_ (M = Fe, Cr) and M_3_O_4_ (M = Fe, Cr) can be detected. The reason for this is that the thickness of the oxidation wear-accumulation layer of the HBHSSs is enhanced, and the multicomponent oxidation layers can form with increased additions of Cr to suppress the occurrence of oxidation wear for HBHSSs.

**Figure 11:**
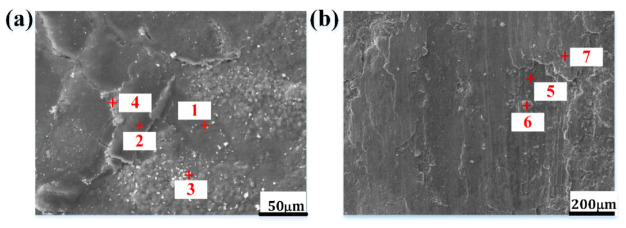
Surface morphologies and EPMA analysis of worn debris for A1 and A4 samples: (**a**) A1, (**b**) A4.

Figure 11 shows the EDX spectrum results of the oxidation wear surface detected by an EPMA instrument. As shown in Figure 11a,b, the selected testing parts are the spallation zones of the oxidation wear debris, where a nearby oxide layer with a little cracks is still intact. The element distribution at each point is shown in Table 3. The content of O element at point 1 is low, which means that the oxide is thin and may likely belong to the Fe-rich oxides (e.g., Fe_2_O_3_ oxide owing to the Fe to O ratio). The color at point 3 in Figure 11a is white and bright, and it contains a small amount of Mo in HBHSSs. This means that the oxide region in point 3, where the alloying elements are easily enriched, may be close to the boundary of the borides and the matrix. Points 2 and 4 in Figure 11a are the oxide scales that should generate the process of peeling off in an oxidation zone with a low O content, as shown with the slight oxidation in the darker parts at point 2 in Figure 11a, while a high O content in brighter parts is detected, indicating serious oxidation. The differences between point 1 and point 3 is that the Ni element locks on, while it only exists in points 2 and 4, meaning that some of the disc materials (i.e., Inconel 718 nickel base superalloy) are adhered and transferred onto the worn surface of the pin sample (i.e., HBHSSs) during the high-temperature oxidation wear process [6,9,12,14]. In Figure 11b, point 5 in sample A4 with a dark color is in the low-O content zone, while the protrusion part at point 6 is bright, with 19.55% O content and high Mo and Mn contents, indicating that point 6 is enriched in oxides and has a high oxidation zone. The color at point 7 is lighter than that of point 5, and the content of O in this area is between points 5 and 6, whereas the content of Ni in this area is very high, which also indicates that part of the disc sample is transferred and adhered onto the worn surface of the pin sample and is overlaid on it to form composite abrasive debris and worn layers. As a matter of fact, in the process of oxidation and spallation, compressive stress may be generated due to the applied loads and growth of oxide scales, which may in turn give rise to the peeling off and fragmentation of generated oxides to produce the composite worn debris adhering to the pin samples. The above surface morphologies and EDX analysis may mean that oxides are mainly involved in the wear process at high temperatures. Undoubtedly, the worn surface of HBHSSs under sliding friction may generate more oxides rapidly acting as the role of abrasives to aggravate further oxidation wear. Obviously, a strong synergistic effect of oxidation and wear, as well as the influence of Cr in the HBHSSs, may alter the oxidation wear rate and wear surface morphologies of HBHSSs.

### 3.5. Wear Mechanism Analysis

Based on the results mentioned above, it is clear that Cr can consolidate the oxidation wear resistance of HBHSSs under different conditions owing the microstructural evolution and interaction between oxidation and wear at elevated temperatures.

**Figure 12:**
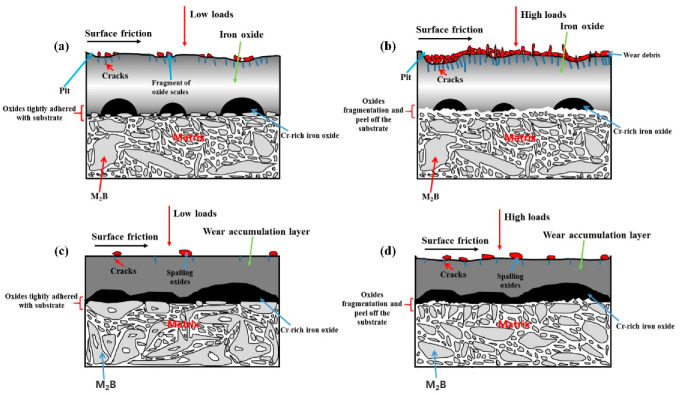
Schematic diagram of high-temperature wear processes and influence of temperatures and loads in HBHSSs: (**a**) low Cr sample under low temperatures and loads; (**b**) low Cr sample under high temperatures and loads; (**c**) high Cr sample under low temperatures and loads; (**d**) high Cr sample under high temperatures and loads.

Figure 12 shows a schematic diagram of the oxidation wear process and the influence of temperatures and loads in HBHSSs. Firstly, the oxides of HBHSSs with low additions of Cr at high elevated temperatures and loads may preferentially occur in the eutectic region of the borides, where the content of Cr is higher owing to the Cr segregation and diffusion during high-temperature wear processes. As shown in Figure 12a, the Fe-rich oxides adjacent to the netlike borides form more easily and can be chipped and cut under the action of the friction force at the contact surface during the wear process. Thus, small pits or broken zones (e.g., cracks or fragmentation of oxide scales) are formed on the worn surface of the samples. As shown in Figure 12b, during the subsequent wear at high temperatures, the worn debris on the surface of HBHSS with low additions of Cr may embed and enter into the pits or cracks, which may accumulate and squeeze into the oxides in the wear layers to produce deformations and further microcracks at higher loads and temperatures. However, high additions of Cr may promote more continuous and compact oxide scales and effectively consolidate the adherence of oxides into the substrate of HBHSSs. At the same time, the hardness of HBHSSs with high additions of Cr can be enhanced to resist harsh high-temperature wear. Therefore, the oxidation wear layer becomes flat and smooth, and serious destruction and fragmentation of the oxidation wear layers are markedly weakened. Meanwhile, the flaking and cracks in the wear layers of HBHSSs are effectively suppressed by improved oxide scales at high temperature, as well as the higher hardness of HBHSSs, as shown in Figure 12c. In other words, the oxidation wear loss of HBHSSs with high additions of Cr is reduced at higher temperatures and loads, as mentioned previously.

It is worth noting that some fragmentation and scattered borides can occur during the prolonged and sustained oxidation wear process at large applied loads; the oxidation wear layers may break down and peel off the substrate, and suffer from a weakened supporting effect of borides in HBHSSs, creating an adverse defending action through which there is no beneficial protection of borides and weak adherence of oxides into the matrix in HBHSSs (Figure 12d). Thus, strong compressive stress and abrasive wear may be generated, causing large damage to oxidation wear layers depending on the synergistic effect of oxidation and wear, as well as the influence of Cr in the HBHSSs. In the cases of high temperatures and large loads, the fragility of the oxide films and cracks in HBHSSs with lower additions of Cr usually takes place. Therefore, some series of factors, including the additions of Cr and temperature, as well as load, may strongly affect the oxidation wear loss of HBHSSs, and the synergistic effect may dominate the oxidation wear process in HBHSSs.

## 4. Conclusions

(1) The as-cast microstructure of HBHSSs is mainly composed of α-Fe, M_2_B (M = Fe,Cr) and a little Fe_3_ (C,B). The addition of Cr results in the transformation of martensite into ferrite and pearlite, and also refines the size of the grid-shaped borides.
(2) After oxidation wear, oxide scales form and the oxidation wear resistance of HBHSSs can be gradually improved with increased additions of Cr. Meanwhile, an interaction between the oxidation wear temperature and applied load on HBHSSs occurs, and the temperature exhibits a larger influence on the oxidation wear properties.
(3) The addition of Cr can reduce the thickness of the uniform and compact oxides and also inhibit the spallation and cracking of worn layers, which is attributed to improvements in microhardness and oxidation resistance of Cr in HBHSSs.
(4) With increased temperatures and loads, more microcracks and spallation of oxides on HBHSSs appear due to a synergistic effect of the temperature and load, which may dominate the oxidation wear process and the failure of oxide films.

## Figures and Tables

**Table 1:** Chemical compositions of cast high-boron high-speed steel analyzed by spark emission spectrometer (wt.%).

Samples	Cr	B	Al	W	Mn	Mo	Si	C	V	Ti
A1	4.80	1.50	1.20	1.20	0.65	0.65	0.60	0.55	0.50	0.08
A2	6.80	1.50	1.20	1.20	0.65	0.65	0.60	0.55	0.50	0.08
A3	8.80	1.50	1.20	1.20	0.65	0.65	0.60	0.55	0.50	0.08
A4	10.80	1.50	1.20	1.20	0.65	0.65	0.60	0.55	0.50	0.08
M2	4.50	—	1.20	5.80	0.23	4.80	0.60	0.90	1.80	0.08

**Table 2:** High-temperature wear test parameters.

Temperature/°C	Load/N	Sliding Speed r/min	Wear Time/min	Gliding Distance/m
600,800	50,100	100	30	236

**Table 3:** Element distribution at each point detected by EPMA in Figure 11 (wt.%).

Point	O	Fe	Cr	Si	Mn	Ni	Mo
1	29.19	66.60	2.64	0.81	0.76	—	—
2	30.18	62.96	3.88	1.07	—	1.91	—
3	36.55	60.63	0.62	0.03	0.74	—	1.42
4	34.47	58.99	3.61	1.37	0.73	0.86	—
5	8.97	72.17	10.53	1.49	2.06	1.32	—
6	19.55	50.28	3.85	—	12.67	—	11.47
7	10.73	32.29	11.78	0.92	1.10	36.20	1.83

## Data Availability

Data sharing is not applicable to this article.
